# iEarth: an interdisciplinary framework in the era of big data and AI for sustainable development

**DOI:** 10.1093/nsr/nwad178

**Published:** 2023-06-24

**Authors:** Peng Gong, Huadong Guo, Bin Chen, Fang Chen, Guojun He, Dong Liang, Zhonghui Liu, Zhongchang Sun, Jin Wu, Zhenci Xu, Dongmei Yan, Hongsheng Zhang

**Affiliations:** Department of Geography, The University of Hong Kong, China; Department of Earth Sciences, The University of Hong Kong, China; Internationl Research Center of Big Data for Sustainable Development Goals, China; Aerospace Information Research Institute, Chinese Academy of Sciences, China; Future Urbanity & Sustainable Environment (FUSE) Lab, Division of Landscape Architecture, Department of Architecture, Faculty of Architecture, The University of Hong Kong, China; Internationl Research Center of Big Data for Sustainable Development Goals, China; Aerospace Information Research Institute, Chinese Academy of Sciences, China; Faculty of Business and Economics, The University of Hong Kong, China; Internationl Research Center of Big Data for Sustainable Development Goals, China; Aerospace Information Research Institute, Chinese Academy of Sciences, China; Department of Earth Sciences, The University of Hong Kong, China; Internationl Research Center of Big Data for Sustainable Development Goals, China; Aerospace Information Research Institute, Chinese Academy of Sciences, China; School of Biological Sciences, The University of Hong Kong, China; Department of Geography, The University of Hong Kong, China; Internationl Research Center of Big Data for Sustainable Development Goals, China; Aerospace Information Research Institute, Chinese Academy of Sciences, China; Department of Geography, The University of Hong Kong, China

## Abstract

The Intelligent Earth (iEarth) framework, composed of four major themes: iEarth data, science, analytics, and decision, is proposed to define and build an interdisciplinary and synergistic framework for research, practice, and education that simultaneously safeguards the sustainable development of our living planet.

The United Nations 2030 Agenda for Sustainable Development provides a shared blueprint for peace and prosperity, addressing environmental, social, and economic challenges through its 17 Sustainable Development Goals (SDGs). Over the past five to seven years, countries worldwide made collective commitments to achieve the SDGs [1]. However, implementing the SDGs agenda faces several challenges, such as insufficient data, unreliable methods, ambiguous indicators, interlinked and constrained targets, diverse localization issues, and other constraints on the pace of progress [2]. The success of the SDGs relies on the coordinated development of environmental, social and economic dimensions, highlighting the urgent need for more transformative and comprehensive countermeasures [3]. This requires the development of an interdisciplinary field that combines natural science, social science and humanities, in a systems-thinking and problem-solving approach.

In 1992, AI Gore introduced the concept of Digital Earth, a digital replica of the entire planet [4]. Since then, the concept has been materialized and repositioned by the pulse from scientific drivers, technological advances, application explorations and community engagements [5]. The growing capacity to collect, store and process multisource data has brought a new transformation enabling digital infrastructure to feed in valuable socio-economic information, making big data an essential resource in the modern digital world [6]. This development has also given rise to Big Earth Data, which envisions the integration of geospatial data from satellites and other Earth observation platforms with socio-economic data, offering geographically contextualized, multi-scale perspectives that help bridge knowledge and information gaps.

The great advancement in collecting Earth, social-economic, and personal characteristic data has tremendous potential if fully used in supporting human society to achieve sustainability, particularly when combined with breakthroughs in AI. However, progress toward integrating such data has lagged far behind the data explosion, and challenges remain in transforming data into information, knowledge and wisdom. To address these issues, we propose an intelligent Earth (iEarth) framework, fueled by Big Earth Data science, which synthesizes interdisciplinary approaches and domain knowledge to quantify Earth system and human civilization processes, reveal complex interactions between natural systems and human society, empower cross-disciplinary ideas and solutions, and generate explicit evidence and valuable scientific knowledge for sustainable development. Upon this wish, we advocate for collaborative and collective efforts within and across boundaries (disciplines, fields, regions, and ethics) to realize an iEarth-powered data-science-analytics-decision support system for sustainable development, encompassing monitoring, evaluation, response and actions.

The concept of iEarth originates from the idea of intelligent Mapping (iMap) [7] and is developed based on a series of disciplinary/interdisciplinary thinking and research studies [8,9]. Here, we define four major themes around the scopes, functions, and implications of iEarth (Fig. 1), including (i) iEarth data, (ii) iEarth science, (iii) iEarth analytics and (iv) iEarth decision.

**Figure 1.**
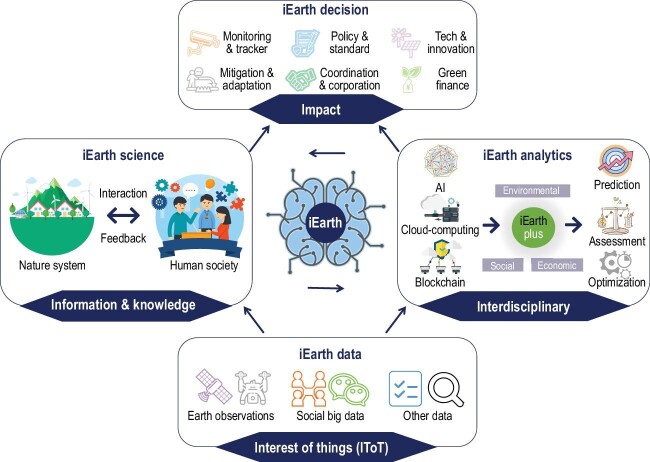
Conceptualized framework of intelligent Earth (iEarth).

*iEarth data.* iEarth data encompasses all Earth system data, including natural systems and human societies, forming the foundation for data-driven analysis and scientific discovery [2]. Regarding quantifying natural systems, Earth observations, the essential element of iEarth data, collect and process historical and real-time data from various complex data sources, including remote sensing, ground observations, internet of things, paleoclimatology data, model simulation (such as CMIP6) and assimilation-based reanalysis. Additionally, the unique space-time characteristic of iEarth data allows for integrating statistical, census, and survey data to quantify the social, economic, and healthy status of human societies, as well as utilizing social sensing big data to quantify human behaviors and mobility patterns.

*iEarth science.* iEarth science is a multidisciplinary subject that examines the natural system, human society, and their

interactions and feedback. The natural system comprises the atmosphere, biosphere, hydrosphere, lithosphere, cryosphere, and related processes that maintain the safety of the Earth's natural environment. Human society, on the other hand, focuses on the socioeconomic context of well-being, covering various aspects of who, what, when, where, why, and how (5W1H) about human activities and human behaviors. Of significant note is the Anthropocene, a proposed epoch dating from the commencement of considerable human impact on Earth's systems, including but not limited to anthropogenic climate change. Particularly, iEarth science aims to bridge the integration of the natural system and human society and advance the fundamental scientific mechanisms underlying the role of human impact (type, magnitude and pattern) on the natural process (response, adaptation, resilience and risk).

*iEarth analytics.* iEarth analytics is the area where the proposed paradigm—‘iEarth plus’ can empower different disciplines and interdisciplinary efforts for SDGs. Leveraging iEarth data and the advanced technologies of artificial intelligence, cloud-computing and blockchain, iEarth analytics aims to embrace proactively different disciplines and fields such as agriculture, engineering, business and economics, sociology, biology, psychology, architecture, medicine, etc., and realize complementary advantages from multidimensional expertise and perspectives. In the era of interdisciplinary collaboration and innovation, this is also a ‘go out and bring in’ strategy for iEarth subject development to be more visible and impactful. As for specifics of iEarth analytics, the general framework includes four key components: monitoring, prediction, assessment and optimization, which can be customized using domain knowledge and interdisciplinary co-design.

*iEarth decision.* iEarth decision supports the implementation of SDGs by monitoring progress, identifying drivers, simulating pathways and evaluating cost-benefits. It is built upon the outcomes from iEarth data, iEarth science and iEarth analytics, to form a data-science-analytics-decision support system for SDGs monitoring, evaluation and response, and to advise spatially and temporally explicit pathways on achieving SDGs.

These four themes are characterized by four ‘Is’ (Fig. 1), respectively, i.e. (i) Interest of things (IToT) for iEarth data, defines the entity and relation of

objective in the Earth system, which contains two essential aspects (entity—anything, or any object detectable, and relation—underlying interaction mechanisms among objects in natural systems and human societies). (ii) Information and knowledge for iEarth science, defines the single or multiple attributes of IToT regarding their spatial, temporal, and contextual information and the underlying mechanisms. (iii) Interdisciplinary for iEarth analytics, defines the visionary paradigm of ‘iEarth plus’ to break disciplinary boundaries and connect Earth observations proactively with other disciplines to reposition the conventional scopes empowered by explicit space, time and context. (iv) Impact for iEarth decision, defines the visible pathways and collective efforts to make differences in advancing domain knowledge, cultivating interdisciplinary innovation, advocating knowledge exchange for implications, and ultimately facilitating the grassroots of the public working together for sustainable development.

Taken together, iEarth is founded on the interplay of the above four major themes (Fig. 1), incorporating multi-source data, cross-disciplinary knowledge, and advanced technology to establish a data-science-analytics-decision support framework (or system) for sustainable environmental, social and economic prosperity (see Supplementary Materials).

The 2030 Agenda specifies 169 targets, measured by 232 unique indicators across 17 SDGs (see https://sdg-tracker.org/). It is exceptionally challenging to secure explicit data and reliable measurements for these complex indicators. To inform this issue, the iEarth framework can support the implementation of SDGs through four main aspects: (i) synthesizing multi-source and multi-attribute datasets to quantify SDGs indicators; (ii) advancing scientific understandings of interaction and feedback between the natural system and human society to uncover limiting factors for SDGs; (iii) coupling interdisciplinary expertise with spatially and temporally explicit monitoring capabilities to realize SDGs evaluation; and (iv) providing decision-making support for guiding SDGs pathways (Fig. 1 and Table S1).

The defining feature of ‘intelligence’ in the iEarth framework is its ability for active learning and knowledge synthesis through AI-powered Big Earth Data models (Fig. S1). Recent advancements in generative pre-training transformer (GPT) have revolutionized natural language processing (NLP) tasks and applications, such as the emerging ChatGPT for natural conversations [10]. In this regard, the AI success in NLP and computer vision (CV) provides unprecedented opportunities to advance, and even revolutionize the paradigm of understanding, modeling, and managing the Earth system and human society. This vision is highly promising due to the massive streams of Earth observations and sensing data reflecting humans’ social, economic, and health status with enhanced spatial and temporal resolutions and multidimensional contexts. Therefore, like the architecture of generative language model, iEarth aims to design and build generative Big Earth Data models that can be pre-trained massively and progressively using text-based and non–text-based data with supervised learning models, reward learning models, and reinforcement learning models (Fig. S1). As a result, Big Earth Data models will provide natural conversation-like responses with explicit data and knowledge, while continuously synthesizing information to improve wisdom in decision making, mitigate social risk, and enhance future resilience for informed iEarth decisions (see Supplementary Materials).

To implement the iEarth framework, both hard and soft infrastructures are critical (see Supplementary Materials), including (i) a robust data infrastructure, which is necessary for effectively collecting, storing, and processing Earth observations, datasets, and information from various complex sources; (ii) a functional and flexible computing infrastructure, expected to complement the physical data infrastructure for transforming data into knowledge; (iii) a financial evaluation, to be incorporated into the decision-support infrastructure, providing cost-benefit analyses for guiding decision making on SDG pathways; and (iv) an open data portal infrastructure, facilitating information visualization and knowledge sharing.

To better consolidate the vision and enhance the capability of iEarth for sustainable development, we have identified key research directions, practice implications and educational curricula (see Supplementary Materials). We aim to define and build an interdisciplinary and synergetic framework for research, practice, and education that simultaneously safeguards the living planet.

## Supplementary Material

nwad178_Supplemental_File
