# Supplementary material for: iEarth: an interdisciplinary framework in the era of big data and AI for sustainable development
Source: Natl Sci Rev. 2023 Jun 24;10(8):nwad178. doi: 10.1093/nsr/nwad178 (PMC10362992; doi:10.1093/nsr/nwad178)
Supplement: nwad178_Supplemental_File [file nwad178_supplemental_file.docx]

**Supplementary Materials for**

iEarth: an interdisciplinary framework in the era of big data and AI for sustainable development

Peng Gong^1,2,*^, Huadong Guo^3,4*^, Bin Chen^5^, Fang Chen^3,4^, Guojun He^6^, Dong Liang^3,4^, Zhonghui Liu^2^ , Zhongchang Sun^3,4^, Jin Wu^7^, Zhenci Xu^1^, Dongmei Yan^3,4^, Hongsheng Zhang^1^

^1^Department of Geography, The University of Hong Kong, Hong Kong, China

^2^Department of Earth Sciences, The University of Hong Kong, Hong Kong, China

^3^Internationl Research Center of Big Data for Sustainable Development Goals, Beijing, China

^4^Aerospace Information Research Institute, Chinese Academy of Sciences, Beijing, China

^5^Future Urbanity & Sustainable Environment (FUSE) Lab, Division of Landscape Architecture, Department of Architecture, Faculty of Architecture, The University of Hong Kong, Hong Kong, China

^6^Faculty of Business and Economics, The University of Hong Kong, Hong Kong, China

^7^School of Biological Sciences, The University of Hong Kong, Hong Kong, China

*Corresponding author: Peng Gong ([penggong@hku.hk](mailto:penggong@hku.hk)), Huadong Guo ([hdguo@radi.ac.cn](mailto:hdguo@radi.ac.cn))

Article type: **Perspective**

**Table of Contents**

**Supplementary Texts**

1. The interaction loops among iEarth data, iEarth science, iEarth analytics and iEarth decision
2. AI opportunities to iEarth science
3. Text-based data in support of iEarth Science
4. Strategies to enhance robustness of Big Earth Data models
5. iEarth hard and soft infrastructure
6. iEarth research, practice, and education

**Supplementary Figure S1**

**Supplementary Table S1**

**Supplementary References**

**Supplementary Texts**

1. **The interaction loops among iEarth data, iEarth science, iEarth analytics and iEarth decision**

iEarth is founded on the interplay of the above four major themes (Figure 1), incorporating multi-source data, cross-disciplinary knowledge, and advanced technology to establish a data-science-analytics-decision support framework (or system) for sustainable environmental, social, and economic prosperity. The interactions among these components can be understood as follows:

1. iEarth Data -> iEarth Science. The process begins with the collection and processing of iEarth data, which comes from diverse sources like remote sensing technologies, in-situ measurements, historical archives, and socio-economic data. This raw data serves as the fundamental input to iEarth science, which aims to understand the complex dynamics of Earth systems and the impact of human activities.
2. iEarth Data -> iEarth Analytics. The iEarth data, gathered from a diverse array of sources, directly informs iEarth analytics. This data is analyzed using computational and statistical models to inform insights about complex Earth system dynamics and interactions between human activities and natural systems.
3. iEarth Science -> iEarth Analytics. The iEarth science, which incorporates theories, models, and empirical understanding of Earth systems and human society, guides the design and application of iEarth analytics. These analytics involve the use of statistical methods, machine learning algorithms, data assimilation techniques, and other computational methods to extract meaningful insights and patterns.
4. iEarth Analytics -> iEarth Science. The insights and patterns identified through iEarth analytics help improve our scientific understanding of Earth systems and human society. iEarth Science not only benefits from the outcomes of analytics but also from its techniques, models, and methods. For example, machine learning or data assimilation techniques used in analytics can be applied or adapted in iEarth science to further understand the physical phenomena and processes that govern Earth systems and human society.
5. iEarth Analytics -> iEarth Decision: The insights generated from iEarth analytics are then used to inform iEarth decisions. These decisions could range from policy choices by governments and international organizations to business strategies by companies and behavior changes by individuals. The decision-making process would ideally involve a thorough understanding of the risks, trade-offs, and uncertainties associated with the analytics' findings.
6. iEarth Science -> iEarth Decision: The scientific understanding developed through iEarth science provides a solid foundation for informed decision-making. The knowledge generated here can guide policy makers, planners, and managers in making decisions that positively impact both human societies and the Earth's systems. These decisions could involve climate policies, land use planning, conservation strategies, and more.”
7. **AI opportunities to iEarth science**

Generative Pretrained Transformer (GPT) models, natural language processing (NLP), and other AI techniques can significantly advance iEarth science in several ways:

1. Integration of multi-source data types. AI can help integrate and analyze diverse types of data, including structured data (e.g., numerical data from sensors) and unstructured data (e.g., text data from scientific literature or social media). This can provide a more comprehensive understanding of Earth systems.
2. Data analysis and interpretation. AI and GPT models can analyze large volumes of data from diverse sources (e.g., satellite data, sensor data, historical records) more efficiently and accurately than traditional methods. It can also handle complex, nonlinear patterns and interactions, which are common in Earth systems.
3. Automated information extraction. NLP models can be used to automatically extract information from scientific literature, reports, and other text sources. This can help researchers stay updated with the latest findings and synthesize knowledge across different studies.
4. Predictive modeling: AI and GPT models can be trained to predict future states of Earth systems based on past and present data. This can improve our ability to forecast weather and climate, predict natural disasters, and manage resources.
5. Causal inference and explanation. With advances in explainable AI, we can use GPT models not just to predict Earth systems but also to understand the underlying causal mechanisms. This can provide valuable insights for managing and optimizing these systems.
6. **Text-based data in support of iEarth Science**

(1) Literature review and meta-analysis. There's a wealth of text-based information in scientific papers, reports, and books. NLP can help automate the process of literature review, extracting essential information from large numbers of documents and summarizing findings across studies.

(2) Geo-tagged Textual Data. Many textual data such as tweets, are associated with specific geographic locations. Analyzing this geo-tagged textual data can provide spatially explicit insights into human activities and perceptions. In addition, social media platforms contain large amounts of other user-generated text data, which can provide insights into public perceptions and behaviors related to environmental issues. This can help in understanding human-nature interactions and inform strategies for behavior change and public engagement.

(3) Sentiment analysis and disaster monitoring. The use of NLP, text analysis, and computational linguistics helps identify and extract subjective information from source materials, which can be used to gauge public opinion on environmental issues or policies. In the meantime, NPL can help analyze real-time social media updates (text-based data) during natural disasters, aiding in disaster response and management.

1. **Strategies to enhance robustness of Big Earth Data models**

The complexities of the Earth system, combined with the potential biases and errors inherent in big data, certainly present challenges for the application of Big Earth Data models in informing decision-making. To address these challenges, here are some possible strategies. (1) Data cleaning and reprocessing. Before using big data for training AI-driven models, it's crucial to conduct thorough data cleaning and preprocessing. This might involve removing or correcting erroneous data, handling missing data, normalizing data, and identifying and mitigating biases in the data. (2) Data integration and fusion. To handle the complexity of Earth systems, it can be beneficial to integrate data from multiple sources and types. This can help capture the diverse aspects and interactions within Earth systems. Techniques such as multi-source data fusion can be used to combine different types of data in a coherent way. (3) Uncertainty quantification. Given the inherent uncertainties in Earth system data and models, it's important to quantify and communicate these uncertainties. Therefore, quantitative approaches like Bayesian methods or ensemble modeling can be used to quantify uncertainty in AI-driven model predictions. (4) Interpretability and explainability. To understand and trust model predictions, it's important to make these models interpretable and explainable. This sometime can involve using simpler, more interpretable models (e.g., decision trees), or applying techniques to make complex models (e.g., deep learning) more interpretable. (5) Physical consistency. To ensure that AI-driven model predictions are physically plausible and consistent with our understanding of Earth systems, it can be beneficial to incorporate physical laws or principles into the models. (6) Model validation and evaluation. To ensure that AI-driven models are accurate and reliable, it's crucial to conduct thorough model validation and evaluation. This might involve cross-validation, and comparing model predictions with independent observations or expert knowledge.

**5**. **iEarth hard and soft infrastructure**

To implement the iEarth framework in the real world through the functional path from iEarth data to iEarth science, iEarth analytics, and iEarth decision, the necessary hard and soft infrastructures are critical.

***(1)*** ***A robust data infrastructure is required to effectively collect, store, and process Earth observations, datasets, and information from various complex sources.*** The challenge lies in integrating different datasets, which have varying data heterogeneities (such as sensors, acquisitions, and formats), organization structure, and spatiotemporal attributes, to generate analysis-ready products. To address this, iEarth aims to develop a unified framework that standardizes, harmonizes, and synthesizes multi-source data ^1^. This framework will provide universal toolkits for data collection, registry, access, integration, and management, as well as processing tools and execution using open geospatial consortium standards. This data standardization and harmonization protocol will ensure the transparency, comparability, and shareability of different remote sensing and social sensing datasets. There have been promising advancements in industries such as Google Earth Engine, Microsoft Planetary Computer, and Earth on Amazon Web Services in terms of planetary-scale applications in the cloud with open geospatial data. However, more attention is needed towards integrating structured Earth observations and non-structured human observations (or social sensing data) in a compatible manner. This is exactly where iEarth aims to take up the challenge and seeks more collective efforts.

***(2)*** ***A functional and flexible computing infrastructure is expected to complement physical data infrastructure to transform data to knowledge.*** On one hand, a suite of processing algorithms (e.g., data quality improvement of radiometric calibration, atmospheric and topographic correction, spatiotemporal filtering, data fusion, etc.), retrieval models (e.g., quantitative parameters of leaf area index, gross primary production, water turbidity, etc.), information extraction methods (e.g., land cover/use types, public infrastructure, socioeconomic status, etc.), prediction modules (e.g., backward and forward projection, spatiotemporal progress and change pattern, etc.), and learning strategies (e.g., ensemble learning, self-supervised learning, transfer learning) will be used to turn the original data to advanced-level data, thematic products, systematic SDG indicators, useful information, and ultimately new knowledge and wisdom. These together define the ‘functional’ feature of the computing infrastructure. On the other hand, iEarth aims to realize a user-oriented flexible computing mode for simulating iEarth solutions in a more computationally efficient way. Different users can define their interest in targets using different contexts of space, time, and attributes ^2^. As a result, they will be accommodated by the relevant data, information, and knowledge within their interests, which is also a good strategy to balance limited computational resources and diverse user services.

***(3)*** ***A financial evaluation should be included in the decision-support infrastructure to provide a cost-benefit analysis for guiding decision-making on SDG pathways.*** This is particularly essential to measure the implementable distance between contemporary situations and targeted SDGs, and the associated investments and efforts required to achieve individual and combined SDGs. Therefore, a financial evaluation module accounting for environmental, social, and economic factors will be the quantitative calculator to compare scenarios for SDG pathways ^3^, ultimately providing different options for decision-makers in a straightforward, comparable, and transparent way. In addition, this financial evaluation module can be driven by short-, medium-, and long-term cost-benefit analysis to provide more reasonable and stepwise solutions for relevant stakeholders’ consideration, given their contextual differences in region, development, ambition, and beneficiaries.

***(4)*** ***An open data portal infrastructure is required to facilitate information visualization and knowledge sharing.*** The iEarth open portal is essential, allowing broader users to dive into any areas of their interests. It not only promotes multi-scale, multidimensional data usages based on the requirements of different spatial and temporal contexts, but also collects feedback on data quality, information reliability, and ground references about SDG progress from crowdsourcing users. In the meantime, informative visualization of data, information, and knowledge to different users is the critical media for promoting knowledge exchange and facilitating real-world actions to close the gap in achieving SDGs worldwide. We believe such a data portal can expedite the distribution and development of iEarth knowledge by providing an easy-to-access, searchable, and interactive hub for various end users such as governments, industries, professionals, relevant stakeholders, and the public.

**6. iEarth research, practice, and education**

**iEarth research.** To better consolidate the vision and enhance the capability of iEarth for supporting SDGs and global environmental change issues, we have identified five key research directions. ***(1) iEarth system observation***. This direction aims to provide essential data and technical support for iEarth system research. This direction includes enriching data infrastructure by collecting, processing, harmonizing, and synthesizing various human-Earth data sources. It also involves exploring innovative technologies for generating new iEarth observations and developing data-model fusion technologies to provide long-term, high-resolution, consistent, and seamless data cubes. ***(2)*** ***iEarth system process and interaction***. This direction focuses on fundamental science studying the laws and mechanisms governing the processes, changes, and responses of the Earth system and its interaction with human activities. This will expand our knowledge pool about the regulation of the natural system, the governance of human society, human-environment interaction and feedback, and how global environmental changes impact these three realms. ***(3) iEarth system analytics and modeling***. This direction aims to empower the quantitative development of iEarth system for both historical reconstruction and future projection using advanced artificial intelligence and high-performance computing technologies. It will work on multi-resolution flexible modeling of the global iEarth system, computable modeling of high-resolution regional and local iEarth system, and the coupled human-environment-health iEarth modeling. ***(4) iEarth plus paradigm for SDGs***. This direction fosters interdisciplinary collaboration and innovation to achieve SDGs. It involves breaking disciplinary boundaries and leveraging complementary strengths to provide iEarth solutions for individual and combined SDGs. It will also reposition short, medium, and long-term SDGs using environmental, social, and economic cost-benefit evaluations and advance the application of the iEarth data-science-analytics-decision support framework locally and globally. ***(5)*** ***iEarth system management for planetary health***. This direction sets the blueprint for adopting new concepts, new paradigms, new theories, and new methods, and combining natural science, social science, economics, and management to promote the future prosperity of our planet. It provides a scientific, technological, humanistic, and economic basis for decision-making on the health of human civilization and the state of the natural systems ^4,5^, which will be guided by multidisciplinary viewpoints and comprehensive assessment methods.

**iEarth practice.** Implementing the iEarth framework impacts a broader spectrum of practices at various scales. The following perspectives of practice are emphasized. ***(1) Effective financial investment by public and private sectors.*** Financial planning and management with strategic investment is vital to the iEarth practice, supporting the entire implementation process. Investment plans will impact the iEarth implementation at different scales. Therefore, governments and private sectors should develop effective and sustainable investment plans locally, nationally, and internationally. ***(2) Stakeholder partnership among governments, industry, and academia.*** Cooperation and partnership are crucial for successfully implementing the iEarth framework to achieve sustainable development across sectors. The partnership is essential in data collection, analytics, modeling, and decision-making processes through various sustainable development pathways. ***(3) Innovative industrial technologies in Big Earth Data and intelligent analysis****.* The industry provides a crucial technological foundation for the iEarth implementation with its innovative intelligent infrastructure, such as cloud computing and high-performance computing platforms (e.g., Google, Microsoft, and Amazon). ***(4) Capacity building by institutes, centers, and laboratories.*** The iEarth implementation for sustainable development creates opportunities for capacity building and development among existing and new institutes, centers, and laboratories. An enhanced iEarth capacity will inherently promote the growth of innovative technology and encourage grassroots implementation in the realm of sustainable development. ***(5) Policies for development frameworks and strategies at different scales.*** The iEarth practice is supported by various policies from different government levels and institutional agreements between stakeholders. Supportive and efficient policies are crucial in ensuring the successful execution of the iEarth framework.

**iEarth education.** iEarth is an interdisciplinary subject that aims to understand intra- and inter-connections among the natural system and human society, and to empower multidimensional expertise and skills to evaluate, respond to, and act towards achieving SDGs. Education institutions play a crucial role in shaping our future ^6^, and embedding iEarth education in curricula through various pedagogies and inspirations is essential for achieving the transformative change needed. A framework for planetary health education has been developed as a common foundational language across disciplines and sectors, serving as a cornerstone for diverse education strategies ^7^. Building on this framework ^6^, iEarth education will focus on six foundational domains representing the essence of iEarth knowledge, technology, practice, and value. (***1)*** ***Earth system and human civilization***. These two themes form the basis for fundamental curriculum development. Earth system is a complex and interconnected system of natural processes that maintain the functioning of the planet, while human civilization has a significant impact on the Earth system through its activities in different forms across space and time. Understanding the interaction and feedback between humans and nature is critical for informing interventions and guiding practical actions. ***(2) Geospatial big data and artificial intelligence.*** These two elements are the powerful leverage of data and technology in tackling complex problems. The integration of big geospatial data and artificial intelligence has the potential to revolutionize our capabilities and efforts to mitigate environmental challenges. Learners should be equipped with technical skillsets in geospatial data collection, data-driven processing, machine learning practices, and computer-aided offline and online visualization. ***(3)*** ***Sustainability and health***. These two aspirations form the core of future development in environmental, social, and economic prosperity. Cross-disciplinary subjects including natural science, social science, economics, and health should be taught to provide a clear understanding of the dimensions of sustainability and the behaviors of health. Through this increasing engagement, learners can create much-needed momentum for mindset change. ***(4) Systems thinking and global vision***. These two intellectual mindsets should be cultivated and enhanced to take the lead in iEarth-driven problem solving. Systems thinking is an interdisciplinary approach that considers the interconnected nature of various components in the coupled human and nature system, and how they interact and influence the entire system. A global vision is essential to broaden learners’ horizons and inspires them to take leadership and responsibility for addressing pressing problems and challenges. ***(5) Governance and management.*** Effective governance and management are critical for realizing sustainable development, as they provide the framework for making decisions and allocating resources that balance social, economic, and environmental considerations. Education in international affairs, governance, public management, and communications are needed to enhance learners’ capabilities in inclusive relationships, optimal strategy, transformational partnerships, and effective communication.


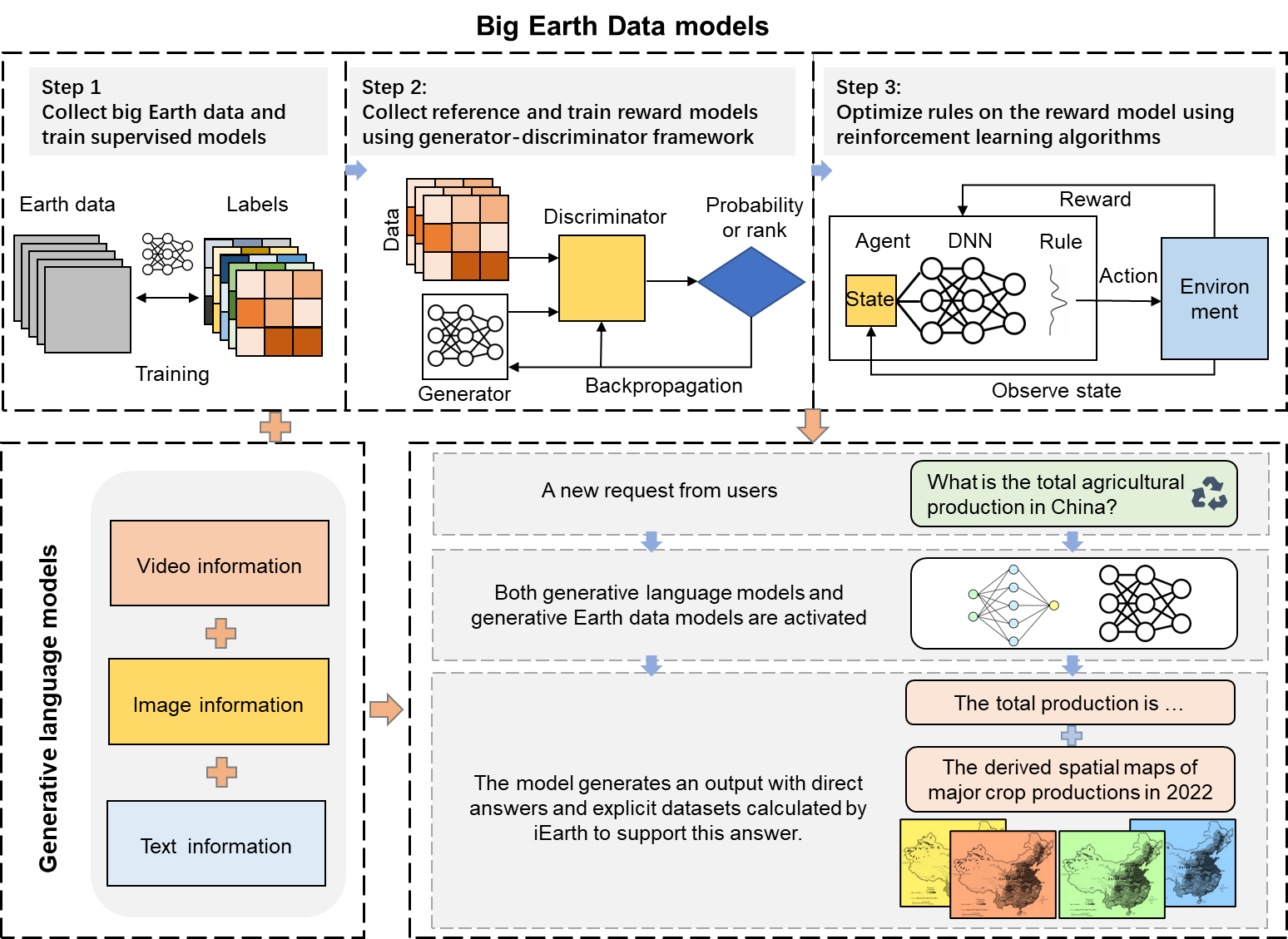


**Figure S1.** The illustrative diagram of active learning and knowledge synthesis through artificial intelligence (AI)-powered Big Earth Data models in the iEarth framework.

**Table S1. Indicative examples of iEarth support for SDGs.**

| SDGs | Exemplary iEarth support for SDGs |
| --- | --- |
| 1. No poverty | Slum detection and socioeconomic status estimate, and associated changes across space and time^8-10^ |
| 1. Zero hunger | Monitoring of cropland distribution, cropping activities, growth status, and yield potential ^11^; Macro-tracking of agricultural supply chains across global, regional, country, and local scales ^12^. |
| 1. Good health and well-being | Monitoring of environmental factors such as air pollution ^13^, thermal condition ^14^, greenspace ^15^, noise pollution ^16^, etc.; Monitoring of human factors such as mobility and behaviors ^17^ to predict disease transmission ^18^ and interpret well-being status ^19^. |
| 1. Quality education | Detection and inventory of educational facilities (i.e., schools, playgrounds, libraries, classrooms, etc.) ^20^ and their nearby open space availability and environmental quality ^21^. |
| 1. Gender equality | Estimation of gender-sensitive facilities (e.g., schools, hospitals, and toilets) ^22^. |
| 1. Clean water and sanitation | Detection and estimation of water quantity ^23,24^ (i.e., 2D coverage and 3D volume) and quality ^25^ (e.g., turbidity, dissolved oxygen volume, and nutrient level), water reserve and purification facilities ^26^, and flooding risk ^27^. |
| 1. Affordable and clean energy | Detection and change analysis of clean energy infrastructure (e.g., solar panels, wind turbines) and conventional power plant facilities ^28,29^. |
| 1. Decent work and economic growth | Monitoring of environmental, social, and economic status for aggregated units/groups and individuals ^30^. |
| 1. Industry, innovation and infrastructure | Inventory and change detection of industrial camps, infrastructures, start-up companies, and patents ^31^. |
| 1. Reduced inequalities | Monitoring and evaluation of environmental injustice ^15,32^ (e.g., green space, thermal threats, air pollution) and socioeconomic inequalities ^33^ (e.g., income, education, health). |
| 1. Sustainable cities and communities | Assessment and monitoring of social, ecological, environmental, and economic dimensions across cities, districts, and communities ^33-36^. |
| 1. Responsible consumption and production | Monitoring of food production ^11,37^ (e.g., cropland distribution and crop yield, livestock, and fishery potential) and consumption ^38^ (e.g., diet and waste). |
| 1. Climate action | Monitoring of climate conditions ^39^ (e.g., temperature, precipitation, extreme weather) and prediction of future climate change ^40,41^. |
| 1. Life below water | Monitoring of marine environment ^42^ (e.g., sea water temperature, acidity) and wildlife ^43^ (e.g., coral reef, fish). |
| 1. Life on land | Monitoring of natural ecosystems (e.g., forest, wildlife habitat, biodiversity) ^40,44,45^. |
| 1. Peace, justice and strong institutions | Surveillance for providing spatially and temporally explicit evidence on justice and law enforcement ^46,47^. |
| 1. Partnerships for the goals | iEarth-based partnership for building observation networks and interdisciplinary think tanks on collective efforts for SDGs ^48^. |

**Supplementary References**

1 Liu, H. *et al.* Production of global daily seamless data cubes and quantification of global land cover change from 1985 to 2020-iMap World 1.0. *Remote Sensing of Environment* **258**, 112364 (2021).

2 Chen, B., Xu, B. & Gong, P. Mapping essential urban land use categories (EULUC) using geospatial big data: Progress, challenges, and opportunities. *Big Earth Data* **5**, 410-441 (2021).

3 Wang, D. *et al.* Economic footprint of California wildfires in 2018. *Nature Sustainability* **4**, 252-260 (2021).

4 Horton, R. *et al.* From public to planetary health: a manifesto. *The Lancet* **383**, 847 (2014).

5 Romanello, M. *et al.* The 2022 report of the Lancet Countdown on health and climate change: health at the mercy of fossil fuels. *The Lancet* **400**, 1619-1654 (2022).

6 Guzmán, C. A. F. *et al.* A framework to guide planetary health education. *The Lancet Planetary Health* **5**, e253-e255 (2021).

7 Stone, S. B., Myers, S. S. & Golden, C. D. Cross-cutting principles for planetary health education. *The Lancet Planetary Health* **2**, e192-e193 (2018).

8 Jean, N. *et al.* Combining satellite imagery and machine learning to predict poverty. *Science* **353**, 790-794 (2016).

9 Pokhriyal, N. & Jacques, D. C. Combining disparate data sources for improved poverty prediction and mapping. *Proceedings of the National Academy of Sciences* **114**, E9783-E9792 (2017).

10 Chi, G., Fang, H., Chatterjee, S. & Blumenstock, J. E. Microestimates of wealth for all low-and middle-income countries. *Proceedings of the National Academy of Sciences* **119**, e2113658119 (2022).

11 Potapov, P. *et al.* Global maps of cropland extent and change show accelerated cropland expansion in the twenty-first century. *Nature Food* **3**, 19-28 (2022).

12 Cottrell, R. S. *et al.* Food production shocks across land and sea. *Nature Sustainability* **2**, 130-137 (2019).

13 Wei, Y. *et al.* NASA Socioeconomic Data and Applications Center (SEDAC), Palisades, New York, (2022).

14 Wu, S., Yu, W. & Chen, B. Observed inequality in thermal comfort exposure and its multifaceted associations with greenspace in United States cities. *Landscape and Urban Planning* **233**, 104701 (2023).

15 Chen, B. *et al.* Contrasting inequality in human exposure to greenspace between cities of Global North and Global South. *Nature Communications* **13**, 1-9 (2022).

16 Maisonneuve, N., Stevens, M. & Ochab, B. Participatory noise pollution monitoring using mobile phones. *Information Polity* **15**, 51-71 (2010).

17 Gonzalez, M. C., Hidalgo, C. A. & Barabasi, A.-L. Understanding individual human mobility patterns. *Nature* **453**, 779-782 (2008).

18 Li, R. *et al.* Substantial undocumented infection facilitates the rapid dissemination of novel coronavirus (SARS-CoV-2). *Science* **368**, 489-493 (2020).

19 Chakrabarti, S., Hamlet, L. C., Kaminsky, J. & Subramanian, S. Association of human mobility restrictions and race/ethnicity–based, sex-based, and income-based factors with inequities in well-being during the COVID-19 pandemic in the United States. *JAMA Network Open* **4**, e217373-e217373 (2021).

20 Graetz, N. *et al.* Mapping local variation in educational attainment across Africa. *Nature* **555**, 48-53 (2018).

21 Dadvand, P. *et al.* Green spaces and cognitive development in primary schoolchildren. *Proceedings of the National Academy of Sciences* **112**, 7937-7942 (2015).

22 Burt, Z., Nelson, K. & Ray, I. *Towards gender equality through sanitation access*. UN WOMEN (2016).

23 Pekel, J.-F., Cottam, A., Gorelick, N. & Belward, A. S. High-resolution mapping of global surface water and its long-term changes. *Nature* **540**, 418 (2016).

24 Zou, Z. *et al.* Divergent trends of open-surface water body area in the contiguous United States from 1984 to 2016. *Proceedings of the National Academy of Sciences* **115**, 3810 (2018).

25 Hou, X. *et al.* Global mapping reveals increase in lacustrine algal blooms over the past decade. *Nature Geoscience* **15**, 130-134 (2022).

26 Lehner, B. *et al.* High‐resolution mapping of the world's reservoirs and dams for sustainable river‐flow management. *Frontiers in Ecology and the Environment* **9**, 494-502 (2011).

27 Tellman, B. *et al.* Satellite imaging reveals increased proportion of population exposed to floods. *Nature* **596**, 80-86 (2021).

28 Kruitwagen, L. *et al.* A global inventory of photovoltaic solar energy generating units. *Nature* **598**, 604-610 (2021).

29 Dunnett, S., Sorichetta, A., Taylor, G. & Eigenbrod, F. Harmonised global datasets of wind and solar farm locations and power. *Scientific Data* **7**, 130 (2020).

30 Yang, L. *et al.* Environmental-social-economic footprints of consumption and trade in the Asia-Pacific region. *Nature Communications* **11**, 4490 (2020).

31 Shi, D., Liu, W. & Wang, Y. Has China’s Young Thousand Talents program been successful in recruiting and nurturing top-caliber scientists? *Science* **379**, 62-65 (2023).

32 Jbaily, A. *et al.* Air pollution exposure disparities across US population and income groups. *Nature* **601**, 228-233 (2022).

33 Pandey, B., Brelsford, C. & Seto, K. C. Infrastructure inequality is a characteristic of urbanization. *Proceedings of the National Academy of Sciences* **119**, e2119890119 (2022).

34 Weiss, D. J. *et al.* A global map of travel time to cities to assess inequalities in accessibility in 2015. *Nature* **553**, 333 (2018).

35 Zhou, Y. *et al.* Satellite mapping of urban built-up heights reveals extreme infrastructure gaps and inequalities in the Global South. *Proceedings of the National Academy of Sciences* **119**, e2214813119 (2022).

36 He, G., Pan, Y. & Tanaka, T. The short-term impacts of COVID-19 lockdown on urban air pollution in China. *Nature Sustainability* **3**, 1005-1011 (2020).

37 Xu, Z. *et al.* Impacts of irrigated agriculture on food–energy–water–CO2 nexus across metacoupled systems. *Nature Communications* **11**, 5837 (2020).

38 Sun, Z. *et al.* Dietary change in high-income nations alone can lead to substantial double climate dividend. *Nature Food* **3**, 29-37 (2022).

39 Findell, K. L. *et al.* The impact of anthropogenic land use and land cover change on regional climate extremes. *Nature Communications* **8**, 989 (2017).

40 Reichstein, M. *et al.* Deep learning and process understanding for data-driven Earth system science. *Nature* **566**, 195-204 (2019).

41 Ravuri, S. *et al.* Skilful precipitation nowcasting using deep generative models of radar. *Nature* **597**, 672-677 (2021).

42 Donlon, C. *et al.* The global ocean data assimilation experiment high-resolution sea surface temperature pilot project. *Bulletin of the American Meteorological Society* **88**, 1197-1214 (2007).

43 Asner, G. P., Martin, R. E. & Mascaro, J. Coral reef atoll assessment in the South China Sea using Planet Dove satellites. *Remote Sensing in Ecology and Conservation* **3**, 57-65 (2017).

44 Wu, J. *et al.* Leaf development and demography explain photosynthetic seasonality in Amazon evergreen forests. *Science* **351**, 972-976 (2016).

45 Yang, R. *et al.* Cost-effective priorities for the expansion of global terrestrial protected areas: Setting post-2020 global and national targets. *Science Advances* **6**, eabc3436 (2020).

46 Hässler, T. *et al.* A large-scale test of the link between intergroup contact and support for social change. *Nature Human Behaviour* **4**, 380-386 (2020).

47 D’Alessandro, S., Cieplinski, A., Distefano, T. & Dittmer, K. Feasible alternatives to green growth. *Nature Sustainability* **3**, 329-335 (2020).

48 Aririguzoh, S. Communication competencies, culture and SDGs: effective processes to cross-cultural communication. *Humanities and Social Sciences Communications* **9**, 1-11 (2022).
